# Impact of symptomatic menopausal transition on the occurrence of depression, anxiety, and sleep disorders: A real-world multi-site study

**DOI:** 10.1192/j.eurpsy.2023.2439

**Published:** 2023-09-12

**Authors:** Dong Yun Lee, Carmen Andreescu, Howard Aizenstein, Helmet Karim, Akiko Mizuno, Antonija Kolobaric, Seokyoung Yoon, Yerim Kim, Jaegyun Lim, Ein Jeong Hwang, Yung-Taek Ouh, Hyung Hoi Kim, Sang Joon Son, Rae Woong Park

**Affiliations:** 1 Department of Biomedical Informatics, Ajou University School of Medicine, Suwon, South Korea; 2 Department of Medical Sciences, Graduate School of Ajou University, Suwon, South Korea; 3 Department of Psychiatry, University of Pittsburgh School of Medicine, Pittsburgh, PA, USA; 4 Department of Bioengineering, University of Pittsburgh, Pittsburgh, PA, USA; 5 Department of Obstetrics and Gynecology, Ajou University School of Medicine, Suwon, South Korea; 6 Department of Neurology, Kangdong Sacred Heart Hospital, Hallym University College of Medicine, Seoul, South Korea; 7 Department of Laboratory Medicine, Myongji Hospital, Hanyang University College of Medicine, Goyang, South Korea; 8 Institute for IT Convergence, Myongji Hospital, Goyang, South Korea; 9 Department of Obstetrics and Gynecology, Graduate School of Medicine, Kangwon National University, Kangwon, South Korea; 10 Department of Laboratory Medicine, Pusan National University Hospital, Busan, South Korea; 11 Department of Psychiatry, Ajou University School of Medicine, Suwon, South Korea; 12 Department of Biomedical Sciences, Ajou University Graduate School of Medicine, Suwon, South Korea

**Keywords:** anxiety, depression, perimenopause, risk factors, sleep disorder

## Abstract

**Background:**

The menopause transition is a vulnerable period that can be associated with changes in mood and cognition. The present study aimed to investigate whether a symptomatic menopausal transition increases the risks of depression, anxiety, and sleep disorders.

**Methods:**

This population-based, retrospective cohort study analysed data from five electronic health record databases in South Korea. Women aged 45–64 years with and without symptomatic menopausal transition were matched 1:1 using propensity-score matching. Subgroup analyses were conducted according to age and use of hormone replacement therapy (HRT). A primary analysis of 5-year follow-up data was conducted, and an intention-to-treat analysis was performed to identify different risk windows over 5 or 10 years. The primary outcome was first-time diagnosis of depression, anxiety, and sleep disorder. We used Cox proportional hazard models and a meta-analysis to calculate the summary hazard ratio (HR) estimates across the databases.

**Results:**

Propensity-score matching resulted in a sample of 17,098 women. Summary HRs for depression (2.10; 95% confidence interval [CI] 1.63–2.71), anxiety (1.64; 95% CI 1.01–2.66), and sleep disorders (1.47; 95% CI 1.16–1.88) were higher in the symptomatic menopausal transition group. In the subgroup analysis, the use of HRT was associated with an increased risk of depression (2.21; 95% CI 1.07–4.55) and sleep disorders (2.51; 95% CI 1.25–5.04) when compared with non-use of HRT.

**Conclusions:**

Our findings suggest that women with symptomatic menopausal transition exhibit an increased risk of developing depression, anxiety, and sleep disorders. Therefore, women experiencing a symptomatic menopausal transition should be monitored closely so that interventions can be applied early.

## Introduction

The menopause transition is a vulnerable time in which women may experience changes in cognition and mood [1, 2]. In self-report surveys conducted in the United Kingdom, half of all menopausal women reported feeling depressed, 37% reported anxiety, 65% reported cognitive impairment, and 64% reported sleep disturbances [3]. The menopause transition is marked by dramatic changes in levels of sex hormones such as oestrogen. These hormonal changes are associated with vasomotor, somatic, cognitive, and mood changes [4]. Specifically, oestrogen is known to interact with serotonin, a neurotransmitter that modulates mood [5, 6]. In addition to serotonin, oestrogen interacts with noradrenaline, a neurotransmitter associated with energy levels, sleep, and arousal [7, 8].

A previous study reported that women who experienced a more symptomatic menopausal transition were at greater risk of depressive symptoms [9]. Moreover, research has demonstrated strong positive associations between symptomatic menopausal transition, which refers to the presence of severe menopausal symptoms during the menopause transition, and mood and anxiety disorders [10]. Although one third of women who experience perimenopause are considered to have symptomatic menopausal transition [11], few studies have focussed on the relationship between symptomatic menopausal transition and mental wellbeing, and their relationship remains unclear [10, 12].

Hormone replacement therapy (HRT) with oestrogen is administered to treat severe menopausal symptoms. HRT is mainly used to relieve vasomotor symptoms, but it may also improve mood symptoms [13, 14]. In the ancillary, Cognitive and Affective Study (KEEPS-Cog) of the Kronos Early Estrogen Prevention Study (KEEPS), women receiving HRT experienced improvements in symptoms of depression and anxiety, but not in cognitive function [15]. However, KEEPS-Cog was limited to women in late menopausal transition and the early postmenopausal periods, and the effect of HRT in women with symptomatic menopausal transition was not examined.

Evaluating the long-term effects of symptoms during the menopause transition may aid in identifying potential factors influencing the development of psychiatric disorders. The current study aimed to characterise the relationship between symptomatic menopausal transition and the risks of depression, anxiety, and sleep disorders. Further, we aimed to evaluate the effects of HRT on the risk of adverse outcomes in women with symptomatic menopausal transition.

### Study design and data sources

We performed a population-based, retrospective observational cohort study using data from approximately 7 million patients across five electronic health record databases in South Korea: Ajou University Hospital (AJH), Kangdong Sacred Heart Hospital (KDH), Myoungji Hospital (MJH), Kangwon University Medical Center (KWMC), and Pusan National University Hospital (PNUH) (see Supplementary Method S1). These databases were standardised using the Observational Medical Outcomes Partnership Common Data Model version 5 [16]. This study was performed using a bio-health big data platform supported by the Korean National Project (https://feedernet.com), which comprises electronic health record data from 37 hospitals (50 million patients) in South Korea.

**Figure 1.**
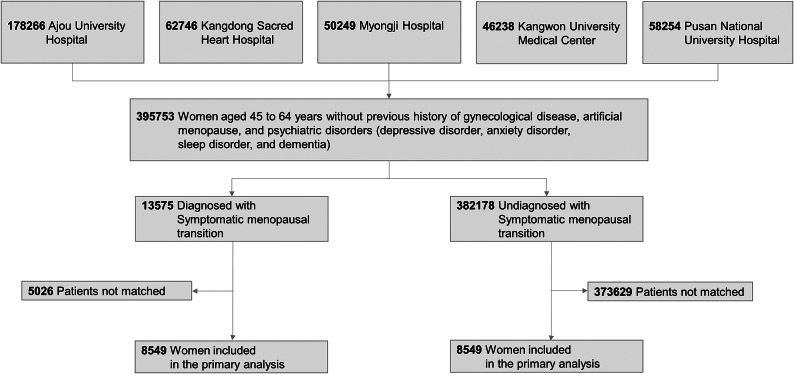
Study flowchart of women aged 45 to 64 years with or without symptomatic menopausal transition.

Symptomatic menopausal transition manifests with many different somatic symptoms, and women who experience symptomatic menopausal transition can be diagnosed with symptomatic menopausal transition [10, 17]. For the current analysis, women diagnosed with symptomatic menopausal transition (ICD-10-CM code: N95) were included in the study cohort, while those without symptomatic menopausal transition were included in the comparison cohort. Symptomatic menopausal transition (N95) included subcodes such as postmenopausal bleeding (N95.0), menopausal and female climacteric states (N95.1), postmenopausal atrophic vaginitis (N95.2), other specified menopausal and perimenopausal disorders (N95.8), and unspecified menopausal and perimenopausal disorder (N95.9). A flow diagram of selection for the study and comparison cohorts is shown in Figure 1. First, we used these databases to identify women aged 45–64 years who were experiencing menopausal transition [12]. We excluded patients with a history of gynaecological diseases (ovarian, endometrial, cervical, and breast cancer) and artificial menopause (hysterectomy, radiotherapy, and chemotherapy). We also excluded patients with a history of psychiatric disorders (depressive disorder, anxiety disorder, and sleep disorder) before the perimenopausal phase. For those without symptomatic menopausal transition, we excluded patients with a history of oestrogen exposure. The date of the first hospital visit at which symptomatic menopausal transition was diagnosed was identified as the index date for each woman in the study cohort, whereas the index date for each woman in the comparison cohort was defined as the date of the earliest hospital visit. Further details of the cohort definitions are presented in Supplementary Method S2.

We conducted distributed network analyses, as described in previous studies [18]. The study package for the entire process was built on the OHDSI Methods Library in R and is available online at https://github.com/ABMI/ImpactOfPMSonMentalDisorder. Each data partner executed this package locally inside the firewall. The pre-designated statistical results (without patient-level information) were then shared for interpretation and database-level meta-analysis. All partners received institutional review board approval or exemption (IRB number: AJIRB-MED-MDB-21-637).

### Outcomes

This study investigated the association between symptomatic menopausal transition diagnosis and the risk of psychiatric disorders. The primary outcome was first-time diagnosis of depression, anxiety, and/or sleep disorder. To increase the diagnostic accuracy, we included all depressive disorders except bipolar depression, all anxiety disorders except phobic disorder, and all sleep disorders except obstructive sleep apnoea and narcolepsy. Furthermore, we applied a restricted definition of the outcomes, which included at least one diagnosis and at least two medication prescriptions (antidepressants for depression, anxiolytics for anxiety disorder, and sleep medications for sleep disorder) any time after the first diagnosis of any of the above psychiatric disorders. Our analysis considered the time-to-first event, with a follow-up duration of 5 years. Further details concerning the outcome definitions are provided in Supplementary Method S3.

### Statistical analyses

We used large-scale propensity score (PS) modelling [19], which included >3,000 baseline patient characteristics between the study and comparison cohorts, including all available demographic characteristics as well as medical, medication, and procedure history in each database. The study populations were then matched using one-to-one greedy matching of the PS. After PS matching, a standardised difference of <0.25 for every covariate was considered negligible [20]. Cox proportional hazard models were used to estimate the association between exposures and outcomes. We then performed a meta-analysis to calculate the summary hazard ratio (HR) estimates across the databases. Before performing the meta-analysis, the *I* ^2^ statistic was used to measure the heterogeneity among the included studies. If the *I* ^2^ value was >50%, a random-effects meta-analysis was performed. If the *I* ^2^ value was <50%, a fixed effects meta-analysis was conducted [21]. A pre-specified two-sided *P-*value of <0.05 was considered statistically significant. We followed the Strengthening the Reporting of Observational Studies in Epidemiology (STROBE) reporting guidelines.

### Sensitivity analyses

Multiple sensitivity analyses were conducted using different definitions of the study population, at-risk time windows, and follow-up duration. We also performed a sensitivity analysis after stratifying the patients according to age (45–54 and 55–64 years) to check if any differences emerged [12]. Additionally, to examine the effects of HRT in patients diagnosed with symptomatic menopausal transition, we compared patients with symptomatic menopausal transition treated with and without HRT. HRT regimens included oestrogen alone as well as oestrogen plus progesterone. Drug exposure was defined as at least 180 days of use in the 3 years after the index date. Furthermore, an additional time-at-risk window (intention-to-treat basis) was applied. The intention-to-treat period was defined as the period from 1 day after the index date until the end of observation. Moreover, to investigate potential surveillance bias, subgroups were stratified according to the follow-up duration (0–1, 1–5, and ≥5 year) since symptomatic menopausal transition diagnosis.

## Results

### Cohort characteristics

A total of 13,575 patients with symptomatic menopausal transition and 382,178 without symptomatic menopausal transition were included across the five data sources. The aggregated patient cohort size, follow-up duration, incidence of psychiatric disorders, and minimum detectable relative risk before and after PS matching in the five databases are shown in Supplementary Table S1.

The baseline characteristics of the overall study population before and after PS matching are shown in Table 1 and Supplementary Table S2. The baseline characteristics before and after PS matching for each database are presented in Supplementary Tables S3–S7. After PS matching, the absolute standardised differences for all baseline characteristics between patients with and without symptomatic menopausal transition were <0.25 within each data source (7,883 pairs in AJH, 6,480 pairs in KDH, 6,095 pairs in MJH, 4,665 pairs in KWMC, and 3,848 pairs in PNUH; see Supplementary Figure S1).

**Table 1.** Baseline characteristics

Patients with and without symptomatic menopausal transition						
	Before matching			After matching		
	With SMT (*n* = 13,575)	Without SMT (*n* = 382,178)	Std. diff.	With SMT (*n* = 8,549)	Without SMT (*n* = 8,549)	Std. diff.
Age (years)						
45**–**49	26.1	38.4	0.30	28.0	30.7	0.07
50**–**54	40.8	23.7	0.51	37.3	37.0	<0.01
55**–**59	22.3	20.2	0.10	22.8	20.9	0.06
60**–**64	10.8	17.6	0.24	11.9	11.4	0.02
Medical history						
Diabetes mellitus	3.1	2.3	0.08	3.4	3.5	<0.01
Hyperlipidaemia	7.4	1.8	0.36	6.4	6.7	<0.01
Obesity	1.8	0.3	0.19	1.8	1.9	<0.01
Cerebrovascular disease	0.7	0.8	0.01	0.7	0.9	0.03
Hypertension	7.7	4.7	0.19	7.5	7.7	0.03
Ischaemic heart disease	0.9	0.9	0.02	1.1	0.9	<0.01
Renal failure syndrome	0.7	0.6	0.03	0.7	0.5	0.03
Chronic liver disease	0.6	0.7	0.02	0.6	0.7	0.02
Urinary tract infection	0.6	0.3	0.06	0.5	0.6	0.04
Malignant neoplastic disease	2.9	3.6	0.02	2.8	3.1	0.03
Medication						
Beta-blocking agents	4.7	3.9	0.06	4.3	4.9	0.06
Calcium channel blocker	5.8	4.5	0.10	5.6	5.8	0.02
Diuretics	5.8	3.6	0.13	5.5	5.7	0.01
ACE inhibitors or angiotensin II antagonist	6.1	3.7	0.18	5.7	5.7	0.01
Anti-thrombotic agents	8.6	7.5	0.06	8.1	8.5	0.01
Blood glucose-lowering drugs	3.4	2.5	0.10	3.6	3.5	0.02
Antibacterial drugs	22.8	17.8	0.14	20.7	22.6	0.07

Values are presented as proportion of patients (%).

Abbreviations: ACE inhibitors, angiotensin-converting enzyme inhibitors; SMT, symptomatic menopausal transition; Std. diff., standardised difference.

### Outcome assessment

**Figure 2.**
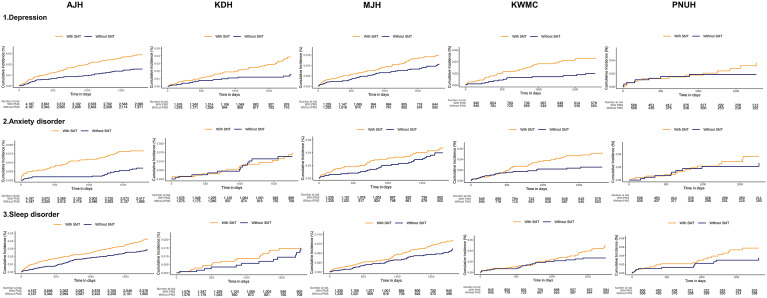
Kaplan–Meier plots for the risk of depression, anxiety, and sleep disorder associated with symptomatic menopausal transition.

**Figure 3.**
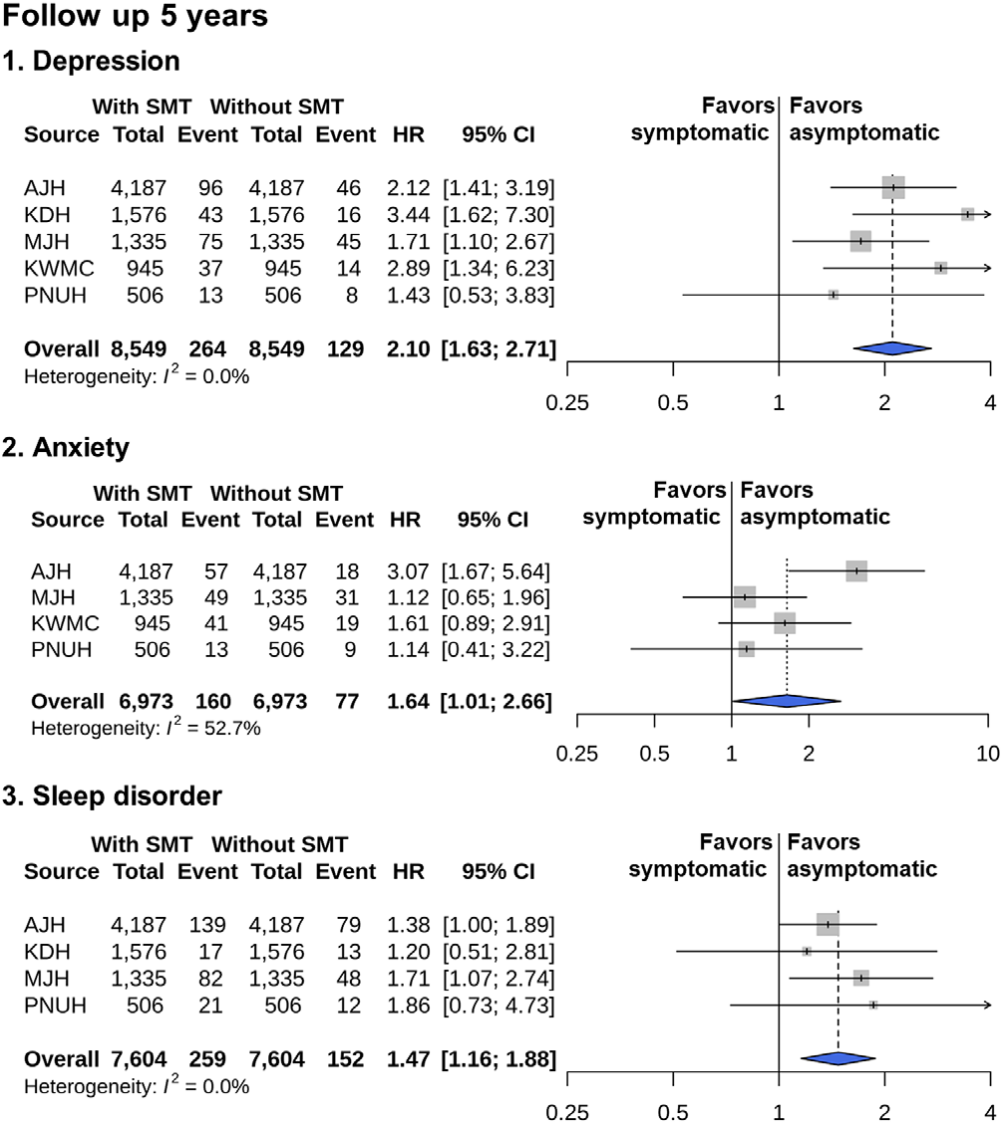
Risk of depression, anxiety, and sleep disorder at the 5-year follow-up.

The survival curves for the occurrence of psychiatric disorders during the 5-year follow-up after PS matching are shown in Figure 2. Cases where the proportional hazards assumption was not met in the survival analysis were excluded from the meta-analysis. Specifically, KDH was excluded for anxiety disorders and KWMC was excluded for sleep disorders. The meta-analytic comparative effect estimates for the outcomes of depressive, anxiety, and sleep disorder are presented in Figure 3. Symptomatic menopausal transition was significantly associated with an increased risk of depression (summary HR 2.10, 95% confidence interval [CI] 1.63–2.10; *P* < 0.01 in meta-analysis). Symptomatic menopausal transition was significantly associated with an increased risk of anxiety (summary HR 1.64, 95% CI 1.01–2.66, *P* = 0.04). Symptomatic menopausal transition was significantly associated with an increased risk of sleep disorder (summary HR 1.47, 95% CI 1.16–1.88, *P* < 0.01).

### Sensitivity analyses

**Figure 4.**
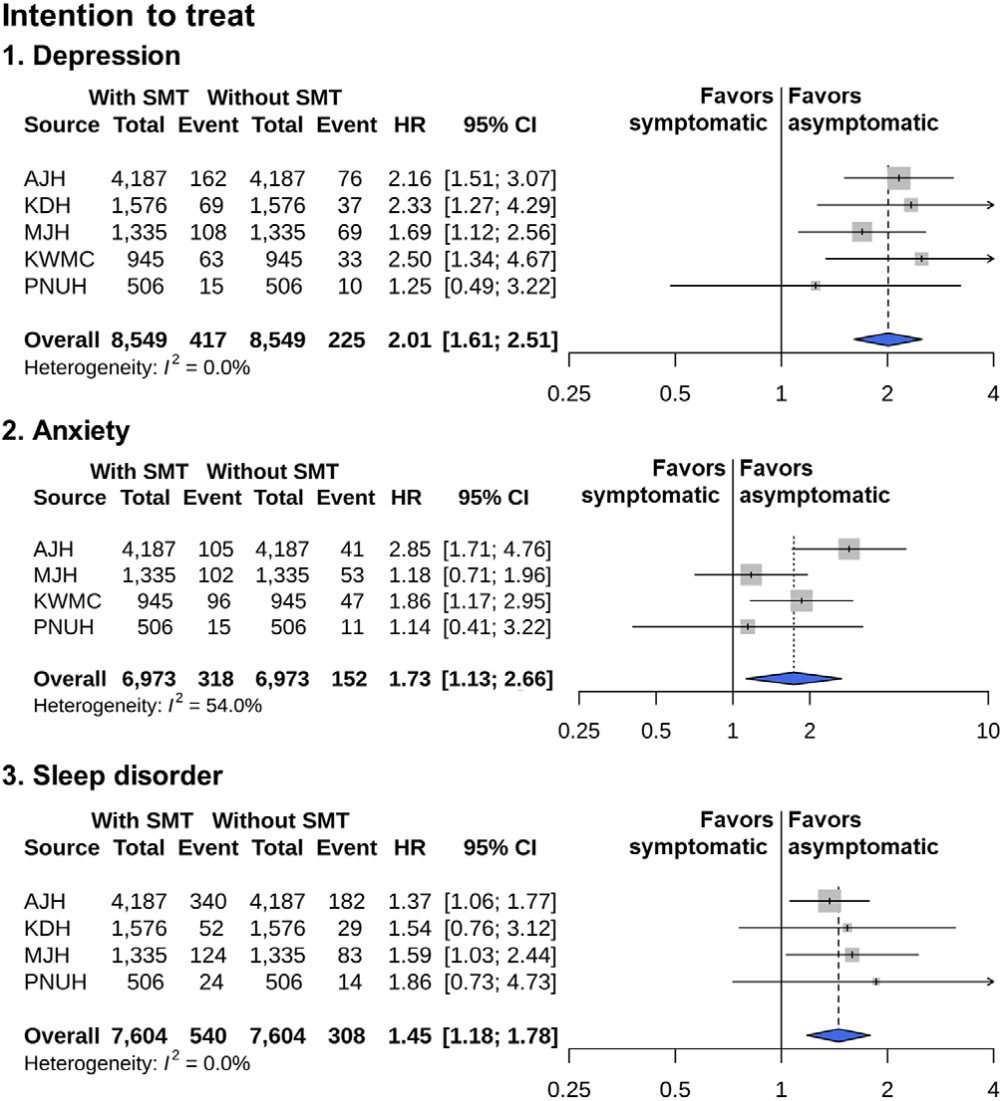
Risk of depression, anxiety, and sleep disorder in intention-to-treat analysis.

The survival curves for the occurrence of psychiatric disorders in the intention-to-treat analysis after PS matching are presented in Supplementary Figure S2. In the different risk windows of the intention-to-treat analysis, symptomatic menopausal transition was significantly associated with an increased risk of depression, anxiety, and sleep disorder (summary HR 2.01, 95% CI 1.61–2.51, *P* < 0.01, summary HR 1.73, 95% CI 1.13–2.66, P = 0.01, and summary HR 1.45, 95% CI 1.18–1.78, *P* < 0.01 in the meta-analysis, respectively; Figure 4).

The meta-analytic comparative effect estimates for the different target age definitions during the 5-year follow-up and intention-to-treat analyses are presented in Supplementary Figures S3 and S4. In the subgroup of patients aged 45–54 years, in both risk windows, symptomatic menopausal transition was significantly associated with an increased risk of depression, anxiety, and sleep disorder (follow-up 5 years: summary HR 1.77, 95% CI 1.33–2.36, *P* < 0.01 in the meta-analysis, summary HR 1.72, 95% CI 1.17–2.55, *P* < 0.01 in the meta-analysis, and summary HR 1.91, 95% CI 1.37–2.66, *P* < 0.01 in the meta-analysis; intention to treat: summary HR 1.62, 95% CI 1.26–2.08, *P* < 0.01 in the meta-analysis, summary HR 1.52, 95% CI 1.09–2.11, *P* = 0.01 in the meta-analysis, and summary HR 1.55, 95% CI 1.20–2.02, *P* < 0.01 in the meta-analysis, respectively) **(**see Supplementary Figure S3). In the subgroup of patients aged 55–64 years, in both risk windows, symptomatic menopausal transition was significantly associated with an increased risk of depression. (5-year follow-up: summary HR 1.73, 95% CI 1.19–2.52, *P* < 0.01 in the meta-analysis; intention to treat: summary HR 1.66, 95% CI 1.19–2.33, *P* < 0.01 in the meta-analysis, respectively) **(**see Supplementary Figure S4). No significance differences related to anxiety disorders and sleep disorders were observed in the subgroup of patients aged 55–64 years.

The meta-analytic comparative effect estimates for HRT in the 5-year follow-up and intention-to-treat analyses are presented in Supplementary Figure S5. At the 5-year follow-up, HRT was significantly associated with an increased risk of depression and sleep disorder (summary HR 2.21, 95% CI 1.07–4.55, *P* = 0.03 in the meta-analysis; summary HR 2.51, 95% CI 1.25–5.04, *P* < 0.01 in the meta-analysis, respectively). In the intention-to-treat analysis, HRT was significantly associated with an increased risk of sleep disorder (summary HR 1.81, 95% CI 1.16–2.82, *P* < 0.01).

Results stratified by follow-up duration are presented in Supplementary Table S8. Incidence rate ratios of depression, anxiety disorder, and sleep disorder significantly increased in all the follow-up duration groups (0–1, 1–5, and 5 years).

## Discussion

In this retrospective cohort study, we estimated the comparative effects of symptomatic menopausal transition on the occurrence of depressive disorder, anxiety disorder, and sleep disorder. Women diagnosed with symptomatic menopausal transition demonstrated associations regarding an increased risk of depressive, anxiety, and sleep disorders when compared with women who had not been diagnosed with symptomatic menopausal transition. In the subgroup of patients aged 45–54 years, symptomatic menopausal transition was significantly associated with an increased risk of depressive disorder, anxiety disorder, and sleep disorder, whereas only the risk of depressive disorder was higher association in the subgroup of patients aged 55–64 years. In the subgroup analysis, for estimating the effect of symptomatic menopausal transition patients with HRT, the use of HRT was associated with an increased risk of sleep disorders when compared with non-use of HRT. These results were consistent for the different risk windows.

Our findings indicating that women diagnosed with symptomatic menopausal transition are associated with an increased risk of depressive disorder are consistent with those of previous studies, including a nationwide cohort study in Taiwan [22]. The presence of clinically significant menopausal symptoms may exacerbate depressive symptoms in perimenopausal women [23]. The pattern for the risk of depression is consistent with the results of our sub-population analysis. Although it has been demonstrated that most women do not develop depression at menopause [24], our results show that perimenopausal symptoms may be a factor in the development of depressive disorders during menopausal transition. Furthermore, since menopausal transition usually lasts for approximately 3–9 years [25], the increased risk of depressive disorder in the sensitivity analysis on an intent-to-treat basis suggests that women who experienced a more symptomatic menopausal transition were at higher risk of developing depression within the 10-year observation period, regardless of whether they currently have menopausal symptoms. This also suggests that in the long-term, lack of protective hormonal effects increases the risk of depression. These findings are consistent with the result of the previous study in which an increased risk of significant depressive symptoms was observed in postmenopausal women [26]. Additionally, considering that sleep quality and anxiety symptoms are positively correlated with perimenopausal symptoms [27, 28], our results regarding the increased risk of anxiety disorders and sleep disorders are in line with previous literature. However, inconsistent results concerning anxiety disorders and sleep disorders were observed in the sub-population analysis. Associations between menopausal symptoms and anxiety/sleep disorders have been less consistent in other studies [28, 29]. In particular, anxiety disorders showed heterogeneity of results even in the total population. Unlike depression and sleep disorders, which have a small number of codes, anxiety disorders have multiple codes with different characteristics, such as panic disorder, mixed depressive anxiety disorder, generalised anxiety disorder, and anxiety disorders caused by drugs or general medical conditions. Due to the variety of anxiety disorder codes, the composition of anxiety disorders may differ between hospitals, which may be related to the heterogeneity of anxiety disorders. It has been reported that there were differences according to institutions for psychiatric patients in South Korea [30]. Overall, the relationship between perimenopausal symptoms and anxiety or sleep disorders may not be as robust as that between perimenopausal symptoms and depression.

In our study, both subgroups by age showed association of an increased risk of depression; however, for anxiety and sleep disorders, only the subgroup of patients aged 45–54 years demonstrated an increased risk. These findings are consistent with the results of epidemiological studies demonstrating that anxiety disorders are more prevalent at younger ages than in the older population [31]. Regarding sleep disorders, their prevalence generally increases with increasing age [32]. However, the prevalence of sleep disorders has been shown to decrease from perimenopause to postmenopause [33]. These results indicate that there is a “window of opportunity” when hormonal imbalance and other perimenopausal changes increase the risk of anxiety and sleep disorders, while for depressive disorders, it appears that the chronic changes conferred by menopause may act as a sustained risk throughout postmenopausal years.

For HRT, the 5-year follow-up results revealed a higher risk of depression in the HRT group, whereas a higher risk of sleep disorders was found in both observation periods. Similar to our findings, Wium-Andersen et al. showed that use of HRT during menopause was associated with risk of depression diagnosis [34]. Consistent with our findings of a higher risk of depression in the first 5 years of HRT, the risk was higher in the first 5 years than in subsequent years. The biological mechanisms linking HRT and depression have suggested that oestradiol has a negative effect on depression and anxiety in the later stages of menopause [35]. Contrary to our findings, Kulkarni et al. showed the antidepressant efficacy of tibolone for depressive disorders in women through the menopause transition [36]. Also, in previous double-blind randomised-controlled trial (RCT), depression rating scale scores were significantly lower in the oestrogen treatment group compared with controls [37]. However, both the Kulkarni and RCT studies had a short observation period of less than 3 months, so the results may be different from a long-term impact evaluation such as our findings. In addition, given that studies have shown that the addition of progesterone can reduce the psychological effects of oestrogen treatment, [38] it is possible that our study design, which included both combination and single agent treatments, had the opposite effect of oestrogen. Since anxiety and depression played a mediating role in the relationship between menopausal symptoms and quality of sleep [27], HRT may have affected sleep through mood symptoms. Another study showed that HRT users in general reported more daytime sleepiness and were more likely to use sleep medications, suggesting an overall poorer quality of sleep [39]. Previous studies have demonstrated that symptom severity during menopausal transition are the main contributors to a poor sleep quality [40]. Association between HRT use and sleep disorder may be because patients receiving HRT had more menopausal symptoms than those without HRT. Our findings on HRT need to be confirmed by studies considering drug composition and symptom severity. Although there is a possibility of bias, our findings do not support previously suggested effects of HRT on depression, anxiety, and sleep disorders [24].

The results of stratification by duration of follow-up indicated that the incidence of depressive disorder, anxiety disorder, and sleep disorder was increased in the first year after the diagnosis of symptomatic menopausal transition. These findings have the potential for surveillance bias. However, the incidence of depressive disorder, anxiety disorder, and sleep disorder remained significantly elevated after the first year of follow-up, suggesting that the observed effects were not solely due to surveillance bias.

In our study, we used a retrospective observational cohort study based on EHR data. Although the RCT is the gold standard, the advantages of the cohort study, such as larger samples and longer follow-up, may provide an alternative to the RCT. Since confounding is a limitation of the cohort study, we applied extensive PS adjustment including 1:1 PS matching to reduce the effects of confounding.

### Limitations

This study had several limitations. First, we could not include psychosocial factors such as stressful life events, attitudes toward menopause, and personality. However, to reduce the effect of a lack of detailed information, we used large-scale PS methods, which were able to balance the unmeasured characteristics [41]. Second, symptomatic menopausal transition may include psychiatric symptoms such as sleep disturbance. Psychiatric or sleep-related menopausal symptoms can also be early signs of psychiatric diseases. However, previous studies have mentioned that most patients are diagnosed with symptomatic menopausal transition based on somatic symptoms, including hot flushes, cold or night sweats, sexual discomfort, vaginal dryness, headaches, and joint pains [22]. Previous papers on the association between menopausal transition and psychiatric disorders have included sleep disorders as an outcome [42]. Third, HRT use was not considered in the primary analysis comparing women with and without symptomatic menopausal transition. However, only 7.7% of women diagnosed with symptomatic menopausal transition received more than 6 months of HRT. Due to the low number of patients treated, patients regardless HRT were used in the primary analysis. Instead, as a sensitivity analysis, we compared differences by HRT status among patients diagnosed with symptomatic menopausal transition. Also, we assumed that only women diagnosed with symptomatic menopausal transition were exposed to HRT and compared the effects of HRT only among women diagnosed with symptomatic menopausal transition. As we assumed, 98.7% of the women who were not diagnosed with symptomatic menopausal transition in our study had never been exposed to HRT during their lifetime. Fourth, in the meta-analysis, the results of Ajou University Hospital contributes all effect sizes for the outcome of anxiety disorders. Especially, when the number of included studies is small (≤5 studies), the results of a meta-analysis should be interpreted with caution [43]. Regarding this, the results should be interpreted with caution, and further analysis using advanced statistical methods may be necessary in the future. Fifth, this study used data from Koreans only; thus, the results cannot be generalized. Sixth, because this was a retrospective cohort study, our findings are only associations and causality cannot be assumed. Seventh, since this was a code-based analysis, data on specific symptoms were not available from the cohorts. For this reason, we were unable to identify if specific symptom types were associated with a greater risk of psychiatric disorders. Further symptom-specific analyses are needed in the future for clinical implications of the relationship between menopausal symptoms and psychiatric disorders. Eighth, it is possible that asymptomatic menopausal women have symptoms but simply choose not to engage in health care. Specifically, there may be instances where patients did not visit their obstetrics and gynaecology despite a physician’s request for a consultation. Our results should be interpreted considering the differences in help-seeking between the asymptomatic and symptomatic groups.

### Conclusion

In conclusion, our findings suggest an increased risk of depression, anxiety, and sleep disorders in women diagnosed with symptomatic menopausal transition. Our study results did not support the therapeutic effects of HRT on depression, anxiety, and sleep disorders. Therefore, more attention should be paid to women with a symptomatic menopausal transition. Additionally, options other than HRT may need to be considered for the treatment of mood symptoms during perimenopause. Considering the number of unmeasured confounders, further investigations are necessary to clarify the association between symptomatic menopausal transition and psychiatric disorders.

## Supporting information

Lee et al. supplementary material Lee et al. supplementary material

Lee et al. supplementary material Lee et al. supplementary material

## Supplementary material

The supplementary material for this article can be found at https://doi.org/10.1192/j.eurpsy.2023.2439.
