# Supplementary material for: Impact of symptomatic menopausal transition on the occurrence of depression, anxiety, and sleep disorders: A real-world multi-site study
Source: Eur Psychiatry. 2023 Sep 12;66(1):e80. doi: 10.1192/j.eurpsy.2023.2439 (PMC10594314; doi:10.1192/j.eurpsy.2023.2439)

**Supplementary material**

**Impact of Symptomatic Menopausal Transition on the Occurrence of Depression, Anxiety, and Sleep Disorders: A Real-World Multi-Site Study**

**Method S1. Data source**

**Ajou University Hospital (AJH)**

The Ajou university hospital (AJH) database is the EHR database of 3 398 600 patients who visited the Ajou University hospital in South Korea from 1994 to 2021. The AUSOM database is in the form of the OMOP-CDM version 5.3.

**Kangdong Sacred Heart Hospital (KDH)**

The Kangdong sacred heart hospital (KDH) database is the EHR database of 1 689 600 patients who visited the Kangdong sacred heart hospital in South Korea from 1986 to 2019. The KDH database is also in the form of the OMOP-CDM version 5.3.

**Myoungji Hospital (MJH)**

The Myoungji hospital (MJH) database is the EHR database of the Myoungji Hospital in South Korea and has data from 882 650 patients who visited the Myoungji Hospital from 2003 to 2021. The MJH database was also included in the OMOP-CDM version 5.3.

**Kangwon University Medical Center (KWMC)**

The Kangwon university medical center (KWMC) database is the EHR database of the Kangwon national university hospital in South Korea and has data from 553 708 patients who visited the hospital from 2003 to 2021. The KWMC database was also included in the OMOP-CDM version 5.3.

**Pusan National University Hospital (PNUH)**

The Pusan national university hospital (PNUH) database is the EHR database of 791 935 patients who visited the Pusan national university hospital in South Korea from 2011 to 2019. The PNUH database is also in the form of the OMOP-CDM version 5.3.

**Method S2. Cohort definitions**

OHDSI’s ATALS is an open source software tool (http://www.ohdsi.org/web/atlas/#/home) for researchers to conduct scientific analyses on standardised observational data converted to the OMOP Common Data model v5.

Researchers can create cohorts by defining groups of people based on an exposure to a drug or diagnosis of a particular condition using healthcare data.

**Women diagnosed with Symptomatic menopausal transition**

We define a cohort of women diagnosed with symptomatic menopausal transition in the following way.

Index rule defining the patient index date:

- A condition occurrence of perimenopausal disorder for the first time in the person’s history with a gender of female and with age between 45 and 64

Inclusion rule based on the index date:

- At least 180 days of observation time prior to the index date
- Limit initial events to: earliest event per person

Exclusion rule based on the index date:

- Previous history of artificial menopause
- Previous history of gynaecological disease
- Previous history of depressive disorder
- Previous history of anxiety disorder
- Previous history of sleep disorder

**Women undiagnosed with Symptomatic menopausal transition**

We define a cohort of Women undiagnosed with symptomatic menopausal transition in the following way.

Index rule defining the patient index date:

- A visit occurrence with a gender of female and with age between 45 and 64

Inclusion rule based on the index date:

- At least 180 days of observation time prior to the index date
- Limit initial events to: earliest event per person

Exclusion rule based on the index date:

- With a condition occurrence of perimenopausal disorder before and after index date
- Previous history of artificial menopause
- Previous history of gynaecological disease
- Previous history of depressive disorder
- Previous history of anxiety disorder
- Previous history of sleep disorder
- Previous history of drug exposure of oestrogen

**Women aged 45-54 diagnosed with Symptomatic menopausal transition**

We define a cohort of women diagnosed with symptomatic menopausal transition in the following way.

Index rule defining the patient index date:

- A condition occurrence of perimenopausal disorder for the first time in the person’s history with a gender of female and with age between 45 and 54

Inclusion rule based on the index date:

- At least 180 days of observation time prior to the index date
- Limit initial events to: earliest event per person

Exclusion rule based on the index date:

- Previous history of artificial menopause
- Previous history of gynaecological disease
- Previous history of depressive disorder
- Previous history of anxiety disorder
- Previous history of sleep disorder

**Women aged 45-54 undiagnosed with Symptomatic menopausal transition**

We define a cohort of Women undiagnosed with symptomatic menopausal transition in the following way.

Index rule defining the patient index date:

- A visit occurrence with a gender of female and with age between 45 and 54

Inclusion rule based on the index date:

- At least 180 days of observation time prior to the index date
- Limit initial events to: earliest event per person

Exclusion rule based on the index date:

- With a condition occurrence of perimenopausal disorder before and after index date
- Previous history of artificial menopause
- Previous history of gynaecological disease
- Previous history of depressive disorder
- Previous history of anxiety disorder
- Previous history of sleep disorder
- Previous history of drug exposure of oestrogen

**Women aged 55-64 diagnosed with Symptomatic menopausal transition**

We define a cohort of women diagnosed with symptomatic menopausal transition in the following way.

Index rule defining the patient index date:

- A condition occurrence of perimenopausal disorder for the first time in the person’s history with a gender of female and with age between 55 and 64

Inclusion rule based on the index date:

- At least 180 days of observation time prior to the index date
- Limit initial events to: earliest event per person

Exclusion rule based on the index date:

- Previous history of artificial menopause
- Previous history of gynaecological disease
- Previous history of depressive disorder
- Previous history of anxiety disorder
- Previous history of sleep disorder

**Women aged 55-64 undiagnosed with Symptomatic menopausal transition**

We define a cohort of Women undiagnosed with symptomatic menopausal transition in the following way.

Index rule defining the patient index date:

- A visit occurrence with a gender of female and with age between 55 and 64

Inclusion rule based on the index date:

- At least 180 days of observation time prior to the index date
- Limit initial events to: earliest event per person

Exclusion rule based on the index date:

- With a condition occurrence of perimenopausal disorder before and after index date
- Previous history of artificial menopause
- Previous history of gynaecological disease
- Previous history of depressive disorder
- Previous history of anxiety disorder
- Previous history of sleep disorder
- Previous history of drug exposure of oestrogen

**Symptomatic menopausal transition patients with hormone replacement therapy**

We define a cohort of women diagnosed with symptomatic menopausal transition in the following way.

Index rule defining the patient index date:

- A condition occurrence of perimenopausal disorder for the first time in the person’s history with a gender of female and with age between 45 and 64

Inclusion rule based on the index date:

- At least 180 days of observation time prior to the index date
- Limit initial events to: earliest event per person
- Drug exposure period of oestrogen is at least 180 days for 3 years after index date

Exclusion rule based on the index date:

- Previous history of artificial menopause
- Previous history of gynaecological disease
- Previous history of depressive disorder
- Previous history of anxiety disorder
- Previous history of sleep disorder

**Symptomatic menopausal transition patients without hormone replacement therapy**

We define a cohort of women diagnosed with symptomatic menopausal transition in the following way.

Index rule defining the patient index date:

- A condition occurrence of perimenopausal disorder for the first time in the person’s history with a gender of female and with age between 45 and 64

Inclusion rule based on the index date:

- At least 180 days of observation time prior to the index date
- Limit initial events to: earliest event per person

Exclusion rule based on the index date:

- With a drug exposure of oestrogen before and after index date
- Previous history of artificial menopause
- Previous history of gynaecological disease
- Previous history of depressive disorder
- Previous history of anxiety disorder
- Previous history of sleep disorder

**Method S3. Outcome definitions**

For each outcome, we developed an operational phenotype definition to determine if observational data could in fact support evaluation of the outcome. We developed definition of outcome cohorts and query to extract them using ATLAS, the OHDSI open-source platform (<https://github.com/OHDSI/atlas>)

**Depressive disorder cohort**

We define an outcome cohort of depressive disorder in the following way.

Index rule defining the patient index date:

- A condition occurrence of depression for the first time in the person’s history

Restrict initial events to having any of the following criteria:

- At least one more condition occurrence of depression after the index date
- At least two drug exposures of antidepressants after the index date
  - Without a condition occurrence of other indications for antidepressants between 30 days before and 7 days after index date
- At least two procedure occurrences of psychiatry procedure after the index date

**Anxiety disorder cohort**

We define an outcome cohort of anxiety disorder in the following way.

Index rule defining the patient index date:

- A condition occurrence of anxiety disorder for the first time in the person’s history

Restrict initial events to having any of the following criteria:

- At least one more condition occurrence of anxiety disorder after the index date
- At least one drug exposure of drugs to treat anxiety disorder after the index date

**Sleep disorder cohort**

We define an outcome cohort of sleep disorder in the following way.

Index rule defining the patient index date:

- A condition occurrence of sleep disorder for the first time in the person’s history

Restrict initial events to having any of the following criteria:

- At least one more condition occurrence of sleep disorder after the index date
- At least one drug exposure of drugs to treat sleep disorder after the index date

**Code list for definitions**

**Perimenopausal disorder**

| OMOP Concept Id | OMOP Concept Name | Domain | Vocabulary | Excluded | Descendants | Mapped |
| --- | --- | --- | --- | --- | --- | --- |
| 4141640 | Perimenopausal disorder | Condition | SNOMED | NO | YES | NO |

**Artificial menopause**

| OMOP Concept Id | OMOP Concept Name | Domain | Vocabulary | Excluded | Descendants | Mapped |
| --- | --- | --- | --- | --- | --- | --- |
| 4029715 | Radiation oncology or radiotherapy | Procedure | SNOMED | NO | YES | NO |
| 4127886 | Hysterectomy | Procedure | SNOMED | NO | YES | NO |
| 4273629 | Chemotherapy | Procedure | SNOMED | NO | YES | NO |

**Gynaecological disease**

| OMOP Concept Id | OMOP Concept Name | Domain | Vocabulary | Excluded | Descendants | Mapped |
| --- | --- | --- | --- | --- | --- | --- |
| 36617644 | Ovarian cancer | Condition | MedDRA | NO | YES | NO |
| 36617575 | Endometrial cancer | Condition | MedDRA | NO | YES | NO |
| 36672483 | Cervical cancer | Condition | MedDRA | NO | YES | NO |
| 36617162 | Breast cancer | Condition | MedDRA | NO | YES | NO |

**Depressive disorder**

| OMOP Concept Id | OMOP Concept Name | Domain | Vocabulary | Excluded | Descendants | Mapped |
| --- | --- | --- | --- | --- | --- | --- |
| 4175329 | Organic mood disorder of depressed type | Condition | SNOMED | NO | YES | NO |
| 440383 | Depressive disorder | Condition | SNOMED | NO | YES | NO |
| 436665 | Bipolar disorder | Condition | SNOMED | YES | YES | NO |
| 442306 | Adjustment disorder with depressed mood | Condition | SNOMED | NO | YES | NO |

**Anxiety disorder**

| OMOP Concept Id | OMOP Concept Name | Domain | Vocabulary | Excluded | Descendants | Mapped |
| --- | --- | --- | --- | --- | --- | --- |
| 4304010 | Phobic disorder | Condition | SNOMED | YES | YES | NO |
| 436074 | Panic disorder | Condition | SNOMED | NO | YES | NO |
| 381537 | Organic anxiety disorder | Condition | SNOMED | NO | YES | NO |
| 4338031 | Mixed anxiety and depressive disorder | Condition | SNOMED | NO | YES | NO |
| 434613 | Generalised anxiety disorder | Condition | SNOMED | NO | YES | NO |
| 4199892 | Anxiety disorder due to a general medical condition | Condition | SNOMED | NO | YES | NO |
| 37109206 | Anxiety disorder caused by drug | Condition | SNOMED | NO | YES | NO |
| 442077 | Anxiety disorder | Condition | SNOMED | NO | NO | NO |

**Sleep disorder**

| OMOP Concept Id | OMOP Concept Name | Domain | Vocabulary | Excluded | Descendants | Mapped |
| --- | --- | --- | --- | --- | --- | --- |
| 435524 | Sleep disorder | Condition | SNOMED | NO | YES | NO |
| 313459 | Sleep apnea | Condition | SNOMED | YES | YES | NO |
| 442588 | Obstructive sleep apnea syndrome | Condition | SNOMED | YES | YES | NO |
| 4102985 | Nonorganic insomnia | Condition | SNOMED | NO | YES | NO |
| 374905 | Non-organic sleep disorder | Condition | SNOMED | NO | YES | NO |
| 436962 | Insomnia | Condition | SNOMED | NO | YES | NO |
| 439708 | Disorders of initiating and maintaining sleep | Condition | SNOMED | NO | YES | NO |
| 437854 | Cataplexy and narcolepsy | Condition | SNOMED | YES | YES | NO |
| 4009650 | Breathing-related sleep disorder | Condition | SNOMED | YES | YES | NO |

**Antidepressants**

| OMOP Concept Id | OMOP Concept Name | Domain | Vocabulary | Excluded | Descendants | Mapped |
| --- | --- | --- | --- | --- | --- | --- |
| 21604686 | Antidepressants | Drug | ATC | NO | YES | NO |

**Other indications for antidepressants**

| OMOP Concept Id | OMOP Concept Name | Domain | Vocabulary | Excluded | Descendants | Mapped |
| --- | --- | --- | --- | --- | --- | --- |
| 440690 | Social phobia | Condition | SNOMED | NO | YES | NO |
| 4242733 | Premenstrual dysphoric disorder | Condition | SNOMED | NO | YES | NO |
| 436074 | Panic disorder | Condition | SNOMED | NO | YES | NO |
| 4175329 | Organic mood disorder of depressed type | Condition | SNOMED | NO | YES | NO |
| 440374 | Obsessive-compulsive disorder | Condition | SNOMED | NO | YES | NO |
| 436962 | Insomnia | Condition | SNOMED | NO | YES | NO |
| 434613 | Generalised anxiety disorder | Condition | SNOMED | NO | YES | NO |
| 4311708 | Diabetic peripheral neuropathy | Condition | SNOMED | NO | YES | NO |
| 438407 | Bulimia nervosa | Condition | SNOMED | NO | YES | NO |
| 436665 | Bipolar disorder | Condition | SNOMED | NO | YES | NO |
| 442306 | Adjustment disorder with depressed mood | Condition | SNOMED | NO | YES | NO |

**Psychiatric procedure**

| OMOP Concept Id | OMOP Concept Name | Domain | Vocabulary | Excluded | Descendants | Mapped |
| --- | --- | --- | --- | --- | --- | --- |
| 45887951 | Psychotherapy Services and Procedures | Procedure | CPT4 | NO | YES | NO |
| 4327941 | Psychotherapy | Procedure | SNOMED | NO | YES | NO |
| 2795675 | Mental Health, Individual Psychotherapy | Procedure | ICD10PCS | NO | YES | NO |
| 2795842 | Mental Health, Electroconvulsive Therapy | Procedure | ICD10PCS | NO | YES | NO |
| 4030840 | Electroconvulsive therapy | Procedure | SNOMED | NO | YES | NO |

**Drugs to treat anxiety**

| OMOP Concept Id | OMOP Concept Name | Domain | Vocabulary | Excluded | Descendants | Mapped |
| --- | --- | --- | --- | --- | --- | --- |
| 743670 | Venlafaxine | Drug | RxNorm | NO | YES | NO |
| 739138 | Sertraline | Drug | RxNorm | NO | YES | NO |
| 722031 | Paroxetine | Drug | RxNorm | NO | YES | NO |
| 724816 | Oxazepam | Drug | RxNorm | NO | YES | NO |
| 702865 | Meprobamate | Drug | RxNorm | NO | YES | NO |
| 791967 | Lorazepam | Drug | RxNorm | NO | YES | NO |
| 777221 | Hydroxyzine | Drug | RxNorm | NO | YES | NO |
| 751412 | Fluvoxamine | Drug | RxNorm | NO | YES | NO |
| 755695 | Fluoxetine | Drug | RxNorm | NO | YES | NO |
| 715939 | Escitalopram | Drug | RxNorm | NO | YES | NO |
| 715259 | Duloxetine | Drug | RxNorm | NO | YES | NO |
| 739323 | Droperidol | Drug | RxNorm | NO | YES | NO |
| 723013 | Diazepam | Drug | RxNorm | NO | YES | NO |
| 717607 | Desvenlafaxine | Drug | RxNorm | NO | YES | NO |
| 790253 | Clorazepate | Drug | RxNorm | NO | YES | NO |
| 798874 | Clonazepam | Drug | RxNorm | NO | YES | NO |
| 19050832 | Clobazam | Drug | RxNorm | NO | YES | NO |
| 797617 | Citalopram | Drug | RxNorm | NO | YES | NO |
| 990678 | Chlordiazepoxide | Drug | RxNorm | NO | YES | NO |
| 733301 | Buspirone | Drug | RxNorm | NO | YES | NO |
| 781039 | Alprazolam | Drug | RxNorm | NO | YES | NO |

**Drugs to treat Sleep disorder**

| OMOP Concept Id | OMOP Concept Name | Domain | Vocabulary | Excluded | Descendants | Mapped |
| --- | --- | --- | --- | --- | --- | --- |
| 744740 | Zolpidem | Drug | RxNorm | NO | YES | NO |
| 704599 | Triazolam | Drug | RxNorm | NO | YES | NO |
| 703547 | Trazodone | Drug | RxNorm | NO | YES | NO |
| 766814 | Quetiapine | Drug | RxNorm | NO | YES | NO |
| 724816 | Oxazepam | Drug | RxNorm | NO | YES | NO |
| 785788 | Olanzapine | Drug | RxNorm | NO | YES | NO |
| 725131 | Mirtazapine | Drug | RxNorm | NO | YES | NO |
| 1301152 | Melatonin | Drug | RxNorm | NO | YES | NO |
| 791967 | Lorazepam | Drug | RxNorm | NO | YES | NO |
| 756349 | Flurazepam | Drug | RxNorm | NO | YES | NO |
| 19055224 | Flunitrazepam | Drug | RxNorm | NO | YES | NO |
| 40254475 | Doxylamine, combinations | Drug | ATC | NO | YES | NO |
| 738156 | Doxepin | Drug | RxNorm | NO | YES | NO |
| 21603454 | Diphenhydramine, combinations | Drug | ATC | NO | YES | NO |
| 1129625 | Diphenhydramine | Drug | RxNorm | NO | YES | NO |
| 723013 | Diazepam | Drug | RxNorm | NO | YES | NO |
| 798874 | Clonazepam | Drug | RxNorm | NO | YES | NO |
| 733301 | Buspirone | Drug | RxNorm | NO | YES | NO |
| 710062 | Amitriptyline | Drug | RxNorm | NO | YES | NO |
| 781039 | Alprazolam | Drug | RxNorm | NO | YES | NO |

**Hormone replacement therapy**

| OMOP Concept Id | OMOP Concept Name | Domain | Vocabulary | Excluded | Descendants | Mapped |
| --- | --- | --- | --- | --- | --- | --- |
| 1548195 | estradiol | Drug | RxNorm | NO | YES | NO |

**Table S1. Patient cohort sizes, primary endpoint events, incidence rates, and minimum detectable relative risk**

**Table S1-1. Depressive disorder**

|  | Patients, n | | PYs, n*year | | Events, n | | IR, /1000PYs | | MDRR |
| --- | --- | --- | --- | --- | --- | --- | --- | --- | --- |
|  | T | C | T | C | T | C | T | C |  |
| Follow up 5 years |  |  |  |  |  |  |  |  |  |
| Before PS matching | 13,575 | 382,178 | 48,172 | 1,091,073 | 440 | 5,006 | 9.13 | 4.59 | 1.23 |
| After PS matching | 8,549 | 8,549 | 30,663 | 26,696 | 264 | 129 | 8.61 | 4.83 | 1.33 |

**Table S1-2. Anxiety disorder**

|  | Patients, n | | PYs, n*year | | Events, n | | IR, /1000PYs | | MDRR |
| --- | --- | --- | --- | --- | --- | --- | --- | --- | --- |
|  | T | C | T | C | T | C | T | C |  |
| Follow up 5 years |  |  |  |  |  |  |  |  |  |
| Before PS matching | 13,575 | 382,178 | 48,555 | 1,094,411 | 306 | 3,882 | 6.30 | 3.55 | 1.27 |
| After PS matching | 8,549 | 8,549 | 30,911 | 26,817 | 176 | 89 | 5.69 | 3.32 | 1.41 |

**Table S1-3. Sleep disorder**

|  | Patients, n | | PYs, n*year | | Events, n | | IR, /1000PYs | | MDRR |
| --- | --- | --- | --- | --- | --- | --- | --- | --- | --- |
|  | T | C | T | C | T | C | T | C |  |
| Follow up 5 years |  |  |  |  |  |  |  |  |  |
| Before PS matching | 13,575 | 382,178 | 48,249 | 1,091,598 | 483 | 5,330 | 10.01 | 4.88 | 1.22 |
| After PS matching | 8,549 | 8,549 | 30,734 | 26,650 | 295 | 171 | 9.60 | 6.42 | 1.30 |

**Table S2. Baseline Characteristics**

| **Patients with and without symptomatic menopausal transition** | | | | | | | | | | |  |
| --- | --- | --- | --- | --- | --- | --- | --- | --- | --- | --- | --- |
|  | Before matching | | |  | After matching | | | | | |  |
|  | With SMT  (n=13 575) | Without SMT  (n=382 178) | P-value |  | | With SMT (n=8 549) | | Without SMT (n=8 549) | | P-value | |
| Age, years |  |  | 0.03* |  |  | |  | | 0.97 | |  |
| 45**–**49 | 26.1 | 38.4 |  |  | 28.0 | | 30.7 | |  | |  |
| 50**–**54 | 40.8 | 23.7 |  |  | 37.3 | | 37.0 | |  | |  |
| 55**–**59 | 22.3 | 20.2 |  |  | 22.8 | | 20.9 | |  | |  |
| 60**–**64 | 10.8 | 17.6 |  |  | 11.9 | | 11.4 | |  | |  |
| Medical history |  |  |  |  |  | |  | |  | |  |
| Diabetes mellitus | 3.1 | 2.3 | 1.00 |  | 3.4 | | 3.5 | | 1.00 | |  |
| Hyperlipidaemia | 7.4 | 1.8 | 0.12 |  | 6.4 | | 6.7 | | 1.00 | |  |
| Obesity | 1.8 | 0.3 | 0.72 |  | 1.8 | | 1.9 | | 1.00 | |  |
| Cerebrovascular disease | 0.7 | 0.8 | 1.00 |  | 0.7 | | 0.9 | | 1.00 | |  |
| Hypertension | 7.7 | 4.7 | 0.56 |  | 7.5 | | 7.7 | | 1.00 | |  |
| Ischemic heart disease | 0.9 | 0.9 | 1.00 |  | 1.1 | | 0.9 | | 1.00 | |  |
| Renal failure syndrome | 0.7 | 0.6 | 1.00 |  | 0.7 | | 0.5 | | 1.00 | |  |
| Chronic liver disease | 0.6 | 0.7 | 1.00 |  | 0.6 | | 0.7 | | 1.00 | |  |
| Urinary tract infection | 0.6 | 0.3 | 1.00 |  | 0.5 | | 0.6 | | 1.00 | |  |
| Malignant neoplastic disease | 2.9 | 3.6 | 1.00 |  | 2.8 | | 3.1 | | 1.00 | |  |
| Medication |  |  |  |  |  | |  | |  | |  |
| Beta blocking agents | 4.7 | 3.9 | 1.00 |  | 4.3 | | 4.9 | | 1.00 | |  |
| Calcium channel blocker | 5.8 | 4.5 | 0.92 |  | 5.6 | | 5.8 | | 1.00 | |  |
| Diuretics | 5.8 | 3.6 | 0.69 |  | 5.5 | | 5.7 | | 1.00 | |  |
| ACE inhibitors or  Angiotensin II antagonist | 6.1 | 3.7 | 0.65 |  | 5.7 | | 5.7 | | 1.00 | |  |
| Anti-thrombotic agents | 8.6 | 7.5 | 0.98 |  | 8.1 | | 8.5 | | 1.00 | |  |
| Blood glucose-lowering drugs | 3.4 | 2.5 | 1.00 |  | 3.6 | | 3.5 | | 1.00 | |  |
| Antibacterial drugs | 22.8 | 17.8 | 0.48 |  | 20.7 | | 22.6 | | 0.88 | |  |

Values are presented as proportion of patients (%). *Indicates statistical significance (P-value < 0.05). Abbreviations: SMT, symptomatic menopausal transition; Std. diff, standardised difference; ACE inhibitors, angiotensin-converting enzyme inhibitors

**Table S3. Baseline characteristics of AJH**

| **Patients With or Without Symptomatic menopausal transition** | | | | | | | |
| --- | --- | --- | --- | --- | --- | --- | --- |
|  | Before matching | | |  | After matching | | |
|  | With SMT  (n= 5 942) | Without SMT  (n=172 324) | Std. diff |  | With SMT (n= 4 187) | Without SMT (n= 4 187) | Std. diff |
| Age, % |  |  |  |  |  |  |  |
| 45-49 | 26.7 | 44.1 | 0.37 |  | 28.9 | 31.7 | 0.06 |
| 50-54 | 43.2 | 23.0 | 0.44 |  | 40.2 | 39.0 | 0.02 |
| 55-59 | 20.9 | 17.6 | 0.08 |  | 20.9 | 19.5 | 0.04 |
| 60-64 | 9.3 | 15.2 | 0.18 |  | 10.0 | 9.8 | 0.01 |
| Medical history, % |  |  |  |  |  |  |  |
| Diabetes mellitus | 3.0 | 2.2 | 0.05 |  | 3.3 | 3.6 | 0.02 |
| Hyperlipidaemia | 7.9 | 1.1 | 0.33 |  | 6.5 | 7.3 | 0.03 |
| Obesity | 3.5 | 0.4 | 0.22 |  | 3.0 | 3.4 | 0.02 |
| Cerebrovascular disease | 0.4 | 0.6 | 0.03 |  | 0.5 | 0.5 | 0.01 |
| Hypertension | 7.7 | 4.8 | 0.12 |  | 7.6 | 7.5 | 0.01 |
| Ischemic heart disease | 0.9 | 1.0 | 0.02 |  | 1.1 | 0.9 | 0.02 |
| Renal failure syndrome | 0.7 | 0.7 | 0.01 |  | 0.7 | 0.5 | 0.03 |
| Chronic liver disease | 0.6 | 0.7 | 0.01 |  | 0.6 | 0.6 | 0.01 |
| Urinary tract infection | 0.5 | 0.3 | 0.03 |  | 0.4 | 0.5 | 0.02 |
| Malignant neoplastic disease | 2.6 | 3.6 | 0.06 |  | 2.5 | 2.4 | 0.01 |
| Medication, % |  |  |  |  |  |  |  |
| Beta blocking agents | 4.1 | 4.0 | 0.01 |  | 4.0 | 4.4 | 0.02 |
| Calcium channel blocker | 6.5 | 5.0 | 0.07 |  | 6.5 | 6.3 | 0.01 |
| Diuretics | 6.4 | 3.7 | 0.12 |  | 6.2 | 6.3 | 0.01 |
| ACE inhibitors or  Angiotensin II antagonist | 6.0 | 3.7 | 0.11 |  | 5.9 | 5.5 | 0.02 |
| Anti-thrombotic agents | 7.4 | 7.4 | 0.01 |  | 7.6 | 7.7 | 0.01 |
| Blood glucose-lowering drugs | 3.3 | 2.5 | 0.05 |  | 3.7 | 3.8 | 0.01 |
| Antibacterial drugs | 21.7 | 18.2 | 0.09 |  | 20.7 | 22.4 | 0.04 |

Values are presented as proportion of the patients (%).

Abbreviations: SMT, symptomatic menopausal transition Std. diff, standardised difference

**Table S4. Baseline characteristics of KDH**

| **Patients With or Without Symptomatic menopausal transition** | | | | | | | |
| --- | --- | --- | --- | --- | --- | --- | --- |
|  | Before matching | | |  | After matching | | |
|  | With SMT  (n= 2 978) | Without SMT  (n=59 768) | Std. diff |  | With SMT (n= 1 576) | Without SMT (n= 1 576) | Std. diff |
| Age, % |  |  |  |  |  |  |  |
| 45-49 | 28.3 | 35.7 | 0.16 |  | 30.2 | 33.1 | 0.06 |
| 50-54 | 36.1 | 25.5 | 0.23 |  | 32.5 | 32.2 | 0.01 |
| 55-59 | 22.8 | 21.6 | 0.03 |  | 23.3 | 21.4 | 0.04 |
| 60-64 | 12.7 | 17.2 | 0.12 |  | 14.0 | 13.3 | 0.02 |
| Medical history, % |  |  |  |  |  |  |  |
| Diabetes mellitus | 4.5 | 2.4 | 0.11 |  | 5.3 | 4.8 | 0.03 |
| Hyperlipidaemia | 3.6 | 1.1 | 0.16 |  | 2.7 | 2.9 | 0.01 |
| Obesity | 0.2 | 0.1 | 0.03 |  | 0.3 | 0.3 | 0.03 |
| Cerebrovascular disease | 0.7 | 1.0 | 0.04 |  | 0.6 | 0.5 | 0.02 |
| Hypertension | 6.6 | 4.0 | 0.12 |  | 6.0 | 5.2 | 0.04 |
| Ischemic heart disease | 0.3 | 0.3 | 0.03 |  | 0.5 | 0.4 | 0.02 |
| Renal failure syndrome | 0.4 | 0.4 | 0.01 |  | 0.4 | 0.5 | 0.01 |
| Chronic liver disease | 0.6 | 0.6 | 0.01 |  | 0.5 | 0.6 | 0.01 |
| Urinary tract infection | 0.3 | 0.2 | 0.01 |  | 0.3 | 0.3 | 0.04 |
| Malignant neoplastic disease | 3.5 | 2.4 | 0.06 |  | 3.4 | 3.7 | 0.02 |
| Medication, % |  |  |  |  |  |  |  |
| Beta blocking agents | 5.3 | 2.6 | 0.14 |  | 4.8 | 5.1 | 0.02 |
| Calcium channel blocker | 5.0 | 3.4 | 0.08 |  | 3.9 | 4.6 | 0.03 |
| Diuretics | 5.3 | 3.4 | 0.09 |  | 4.3 | 5.1 | 0.04 |
| ACE inhibitors or  Angiotensin II antagonist | 5.3 | 2.3 | 0.16 |  | 4.1 | 4.4 | 0.02 |
| Anti-thrombotic agents | 10.2 | 5.8 | 0.16 |  | 8.8 | 8.9 | 0.01 |
| Blood glucose-lowering drugs | 3.2 | 2.1 | 0.07 |  | 2.5 | 2.5 | 0.01 |
| Antibacterial drugs | 19.7 | 16.0 | 0.10 |  | 16.6 | 18.7 | 0.06 |

**Table S5. Baseline characteristics of MJH**

| **Patients With or Without Symptomatic menopausal transition** | | | | | | | |
| --- | --- | --- | --- | --- | --- | --- | --- |
|  | Before matching | | |  | After matching | | |
|  | With SMT  (n= 2 354) | Without SMT  (n=47 895) | Std. diff |  | With SMT (n= 1 335) | Without SMT (n= 1 335) | Std. diff |
| Age, % |  |  |  |  |  |  |  |
| 45-49 | 26.2 | 40.5 | 0.31 |  | 28.8 | 32.7 | 0.08 |
| 50-54 | 40.3 | 24.1 | 0.35 |  | 35.1 | 36.1 | 0.02 |
| 55-59 | 21.7 | 19.2 | 0.06 |  | 22.7 | 19.4 | 0.08 |
| 60-64 | 11.7 | 16.2 | 0.13 |  | 13.3 | 11.8 | 0.05 |
| Medical history, % |  |  |  |  |  |  |  |
| Diabetes mellitus | 2.6 | 1.4 | 0.09 |  | 2.9 | 3.4 | 0.03 |
| Hyperlipidaemia | 11.5 | 3.5 | 0.31 |  | 10.7 | 9.1 | 0.06 |
| Obesity | 1.1 | 0.3 | 0.10 |  | 1.0 | 0.7 | 0.04 |
| Cerebrovascular disease | 1.2 | 0.7 | 0.05 |  | 1.0 | 1.2 | 0.02 |
| Hypertension | 10.2 | 5.4 | 0.18 |  | 9.8 | 9.0 | 0.03 |
| Ischemic heart disease | 2.1 | 1.1 | 0.08 |  | 2.1 | 1.8 | 0.02 |
| Renal failure syndrome | 0.8 | 0.4 | 0.05 |  | 0.8 | 0.6 | 0.03 |
| Chronic liver disease | 0.7 | 0.4 | 0.04 |  | 0.5 | 0.4 | 0.01 |
| Urinary tract infection | 1.1 | 0.4 | 0.08 |  | 0.8 | 1.0 | 0.02 |
| Malignant neoplastic disease | 1.7 | 0.8 | 0.08 |  | 1.3 | 2.2 | 0.07 |
| Medication, % |  |  |  |  |  |  |  |
| Beta blocking agents | 4.5 | 3.3 | 0.06 |  | 4.4 | 4.7 | 0.01 |
| Calcium channel blocker | 6.5 | 4.0 | 0.11 |  | 6.3 | 6.0 | 0.01 |
| Diuretics | 7.1 | 3.4 | 0.17 |  | 6.6 | 6.2 | 0.02 |
| ACE inhibitors or  Angiotensin II antagonist | 6.8 | 3.4 | 0.15 |  | 6.4 | 5.8 | 0.02 |
| Anti-thrombotic agents | 10.4 | 5.9 | 0.17 |  | 9.3 | 10.9 | 0.05 |
| Blood glucose-lowering drugs | 4.1 | 1.8 | 0.14 |  | 4.2 | 4.1 | 0.01 |
| Antibacterial drugs | 30.5 | 16.5 | 0.34 |  | 27.3 | 29.6 | 0.05 |

**Table S6. Baseline characteristics of KWMC**

| **Patients With or Without Symptomatic menopausal transition** | | | | | | | |
| --- | --- | --- | --- | --- | --- | --- | --- |
|  | Before matching | | |  | After matching | | |
|  | With SMT  (n= 1 691) | Without SMT  (n=44 547) | Std. diff |  | With SMT (n= 945) | Without SMT (n= 945) | Std. diff |
| Age, % |  |  |  |  |  |  |  |
| 45-49 | 24.9 | 37.7 | 0.28 |  | 27.1 | 28.4 | 0.03 |
| 50-54 | 43 | 24.8 | 0.39 |  | 36.1 | 38.7 | 0.06 |
| 55-59 | 23.1 | 19.6 | 0.09 |  | 25.4 | 22.8 | 0.06 |
| 60-64 | 9.1 | 17.8 | 0.26 |  | 11.4 | 10.2 | 0.04 |
| Medical history, % |  |  |  |  |  |  |  |
| Diabetes mellitus | 0.5 | 0.3 | 0.03 |  | 0.7 | 0.5 | 0.03 |
| Hyperlipidaemia | 7.1 | 2.0 | 0.25 |  | 6.8 | 8.7 | 0.07 |
| Obesity | 0.3 | 0.1 | 0.03 |  | 0.5 | 0.5 | 0.05 |
| Cerebrovascular disease | 1.0 | 0.7 | 0.03 |  | 1.2 | 2.1 | 0.08 |
| Hypertension | 4.8 | 2.6 | 0.12 |  | 5.2 | 7.1 | 0.08 |
| Ischemic heart disease | 0.3 | 0.6 | 0.04 |  | 0.5 | 0.7 | 0.06 |
| Renal failure syndrome | 0.3 | 0.3 | 0.03 |  | 0.5 | 0.5 | 0.03 |
| Chronic liver disease | 0.3 | 0.3 | 0.03 |  | 0.5 | 0.7 | 0.10 |
| Urinary tract infection | 0.9 | 0.4 | 0.07 |  | 0.7 | 0.8 | 0.01 |
| Malignant neoplastic disease | 0.8 | 0.8 | 0.01 |  | 0.8 | 1.3 | 0.04 |
| Medication, % |  |  |  |  |  |  |  |
| Beta blocking agents | 5.9 | 3.2 | 0.13 |  | 4.9 | 5.9 | 0.05 |
| Calcium channel blocker | 3.8 | 2.2 | 0.09 |  | 3.1 | 3.7 | 0.04 |
| Diuretics | 3.9 | 2.3 | 0.10 |  | 3.6 | 3.9 | 0.02 |
| ACE inhibitors or  Angiotensin II antagonist | 5.5 | 2.7 | 0.14 |  | 4.6 | 5.9 | 0.06 |
| Anti-thrombotic agents | 7.7 | 6.6 | 0.04 |  | 7.2 | 8.0 | 0.03 |
| Blood glucose-lowering drugs | 2.6 | 1.7 | 0.06 |  | 3.1 | 3.3 | 0.01 |
| Antibacterial drugs | 23.2 | 15.4 | 0.20 |  | 20.6 | 24.0 | 0.08 |

**Table S7. Baseline characteristics of PNUH**

| **Patients With or Without Symptomatic menopausal transition** | | | | | | | |
| --- | --- | --- | --- | --- | --- | --- | --- |
|  | Before matching | | |  | After matching | | |
|  | With SMT  (n= 610) | Without SMT  (n=57 644) | Std. diff |  | With SMT (n= 506) | Without SMT (n= 506) | Std. diff |
| Age, % |  |  |  |  |  |  |  |
| 45-49 | 12.7 | 23.1 | 0.27 |  | 13.7 | 14.5 | 0.02 |
| 50-54 | 37 | 23 | 0.31 |  | 35.5 | 34.3 | 0.02 |
| 55-59 | 32.8 | 27.7 | 0.11 |  | 32.7 | 32.1 | 0.01 |
| 60-64 | 17.5 | 26.2 | 0.21 |  | 18.1 | 19.1 | 0.03 |
| Medical history, % |  |  |  |  |  |  |  |
| Diabetes mellitus | 5.5 | 4.7 | 0.04 |  | 5.3 | 4.3 | 0.04 |
| Hyperlipidaemia | 5.7 | 2.9 | 0.14 |  | 5.2 | 3.8 | 0.07 |
| Obesity | 0.8 | 0.1 | 0.10 |  | 1.0 | 1.0 | 0.07 |
| Cerebrovascular disease | 1.1 | 1.2 | 0.01 |  | 1.0 | 2.2 | -0.1 |
| Hypertension | 11.3 | 6.1 | 0.18 |  | 10.0 | 14.3 | 0.13 |
| Ischemic heart disease | 1.0 | 1.6 | 0.06 |  | 1.0 | 1.2 | 0.02 |
| Renal failure syndrome | 1.8 | 0.9 | 0.08 |  | 1.4 | 1.0 | 0.06 |
| Chronic liver disease | 2.0 | 1.5 | 0.04 |  | 2.0 | 2.4 | 0.03 |
| Urinary tract infection | 1.0 | 0.3 | 0.08 |  | 1.0 | 1.4 | 0.08 |
| Malignant neoplastic disease | 13.1 | 9.1 | 0.12 |  | 11.2 | 12.9 | 0.05 |
| Medication, % |  |  |  |  |  |  |  |
| Beta blocking agents | 4.9 | 5.7 | 0.04 |  | 4.4 | 7.2 | 0.12 |
| Calcium channel blocker | 6.5 | 6.3 | 0.01 |  | 7.0 | 8.6 | 0.06 |
| Diuretics | 3.6 | 4.5 | 0.05 |  | 3.4 | 4.4 | 0.05 |
| ACE inhibitors or  Angiotensin II antagonist | 9.8 | 5.9 | 0.15 |  | 9.8 | 11.2 | 0.05 |
| Anti-thrombotic agents | 9.0 | 11.6 | 0.09 |  | 8.2 | 9.0 | 0.03 |
| Blood glucose-lowering drugs | 5.9 | 4.2 | 0.08 |  | 5.4 | 3.4 | 0.1 |
| Antibacterial drugs | 9.3 | 11.0 | 0.06 |  | 11.8 | 11.3 | 0.02 |

**Table S8. Number of Newly Diagnosed Depressive, Anxiety, and Sleep Disorders Between Women With and Without Symptomatic Menopausal Transition Which Was Stratified by Follow-Up Duration** **at intention-to-treat**

| **Patients with and without symptomatic menopausal transition** | | | | | |
| --- | --- | --- | --- | --- | --- |
|  | With SMT | | Without SMT | |  |
| Follow-Up Duration (years) | Number of outcomes | Per 1000 Person-Years | Number of outcomes | Per 1000 Person-Years | Risk Ratio (95% CI) |
| Depression | | | | | |
| Overall | 417 | 8.69 | 225 | 5.56 | 2.01*  [1.61; 2.51] |
| 0-1 | 98 | 186.89 | 53 | 76.78 | 2.76*  [1.99; 3.83] |
| 1-5 | 166 | 23.05 | 75 | 10.51 | 2.17*  [1.66; 2.83] |
| ≥5 | 153 | 3.80 | 97 | 2.97 | 1.29*  [1.01; 1.66] |
| Anxiety disorder | | | | | |
| Overall | 318 | 8.03 | 152 | 4.54 | 1.73*  [1.13; 2.66] |
| 0-1 | 59 | 144.93 | 29 | 53.12 | 3.07*  [1.98; 4.76] |
| 1-5 | 101 | 16.81 | 48 | 7.95 | 2.12*  [1.51; 2.97] |
| ≥5 | 158 | 4.76 | 75 | 2.79 | 1.70*  [1.30; 2.23] |
| Sleep disorder | | | | | |
| Overall | 540 | 12.79 | 308 | 8.73 | 1.45*  [1.18; 1.78] |
| 0-1 | 94 | 196.82 | 45 | 70.69 | 3.10*  [2.19; 4.40] |
| 1-5 | 165 | 25.34 | 107 | 16.48 | 1.54*  [1.22; 1.95] |
| ≥5 | 281 | 7.97 | 156 | 5.54 | 1.43*  [1.18; 1.73] |

Abbreviations: SMT, symptomatic menopausal transition; CI, confidence interval

*Statistical significance

**Figure S1. Covariate balance plot for comparing the risk of depressive disorder between women with SMT and without SMT before and after propensity score matching**


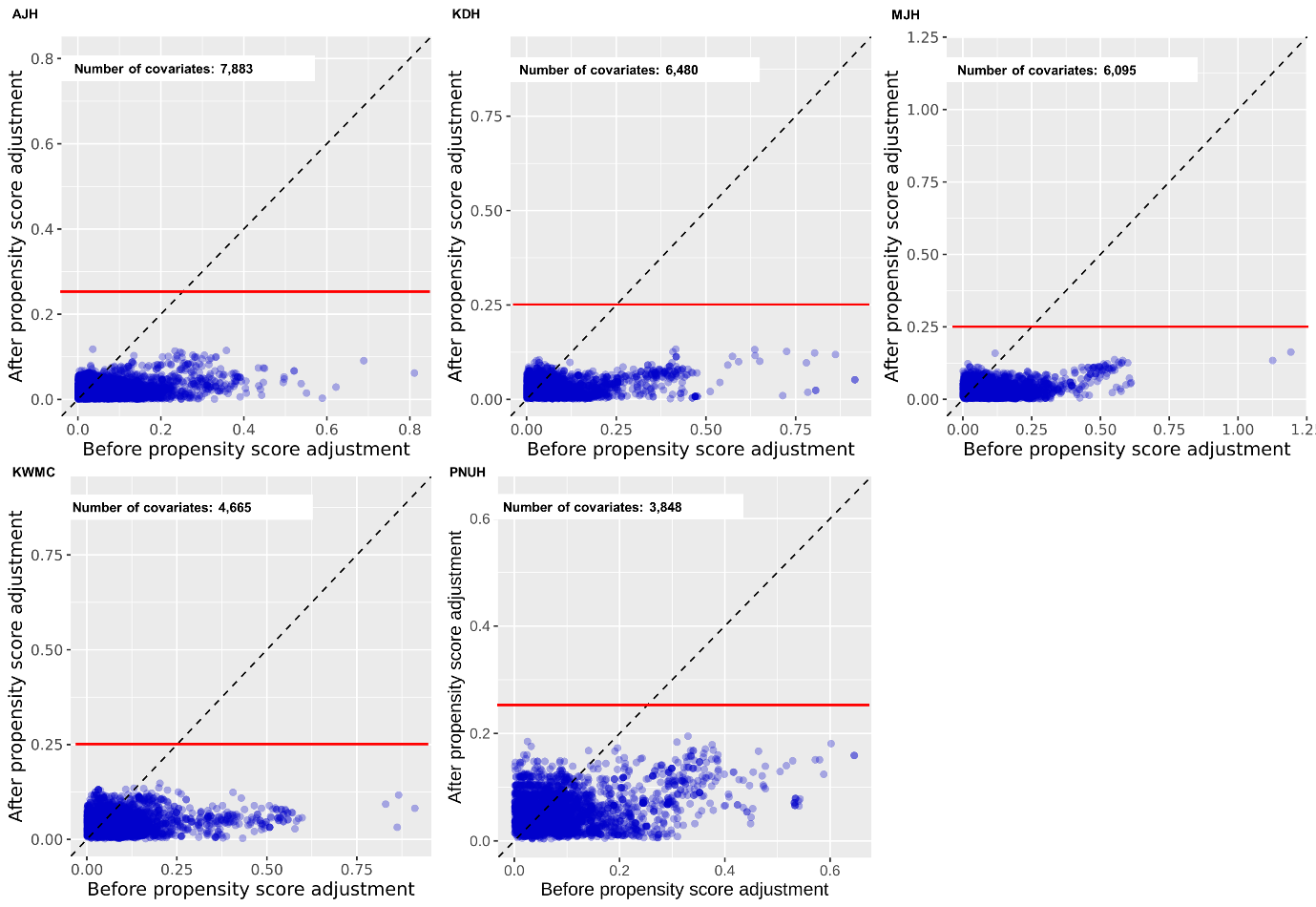


Abbreviations: SMT, symptomatic menopausal transition

**Figure S2 Kaplan-Meier plots for the risks of depression, anxiety, and sleep disorder associated with Symptomatic menopausal transition at intention-to-treat**


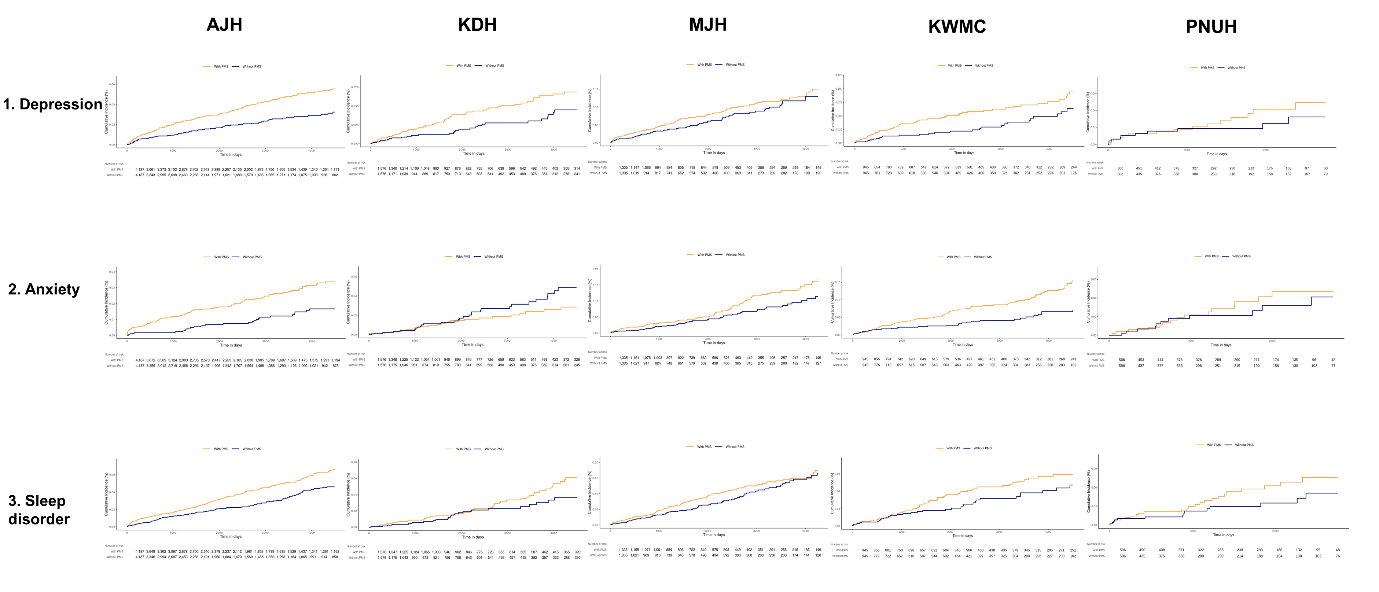


**Figure S3 Sensitive analyses for risks of outcomes associated with Symptomatic menopausal transition, analysed using study population aged 45-54**


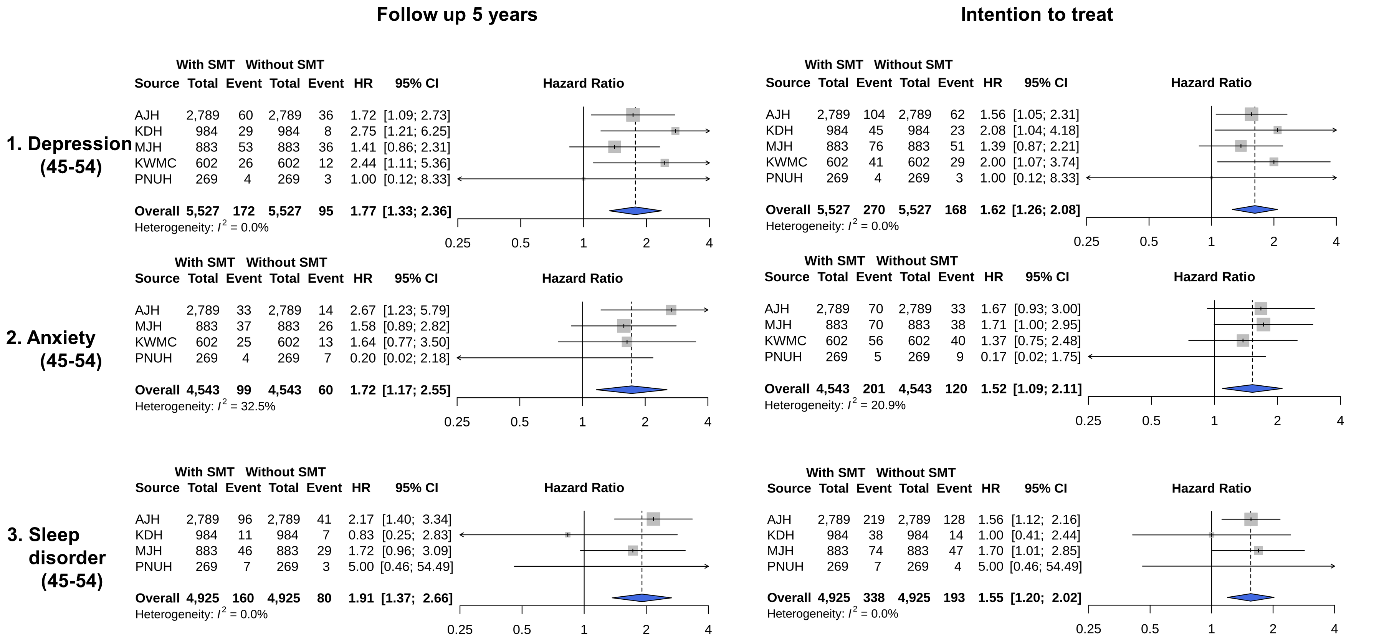


**Figure S4 Sensitive analyses for risks of outcomes associated with Symptomatic menopausal transition, analysed using study population aged 55-64**


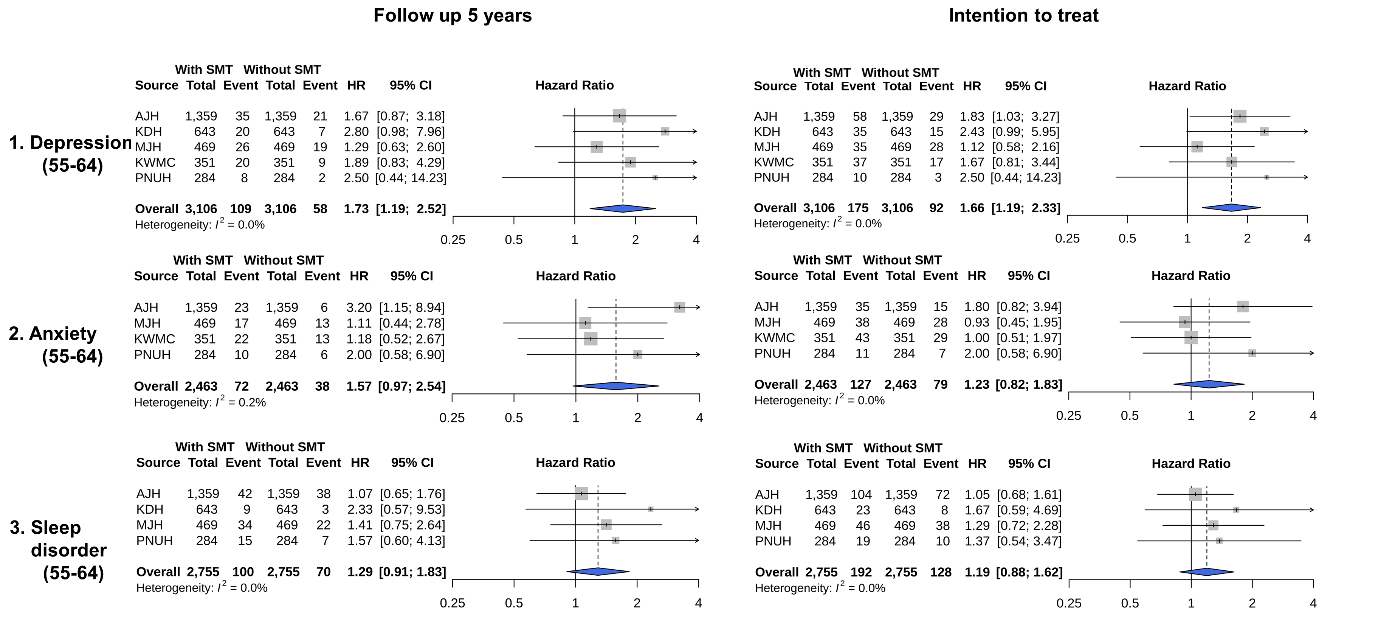


**Figure S5 Sensitive analyses for risks of outcomes associated with hormone replacement therapy**


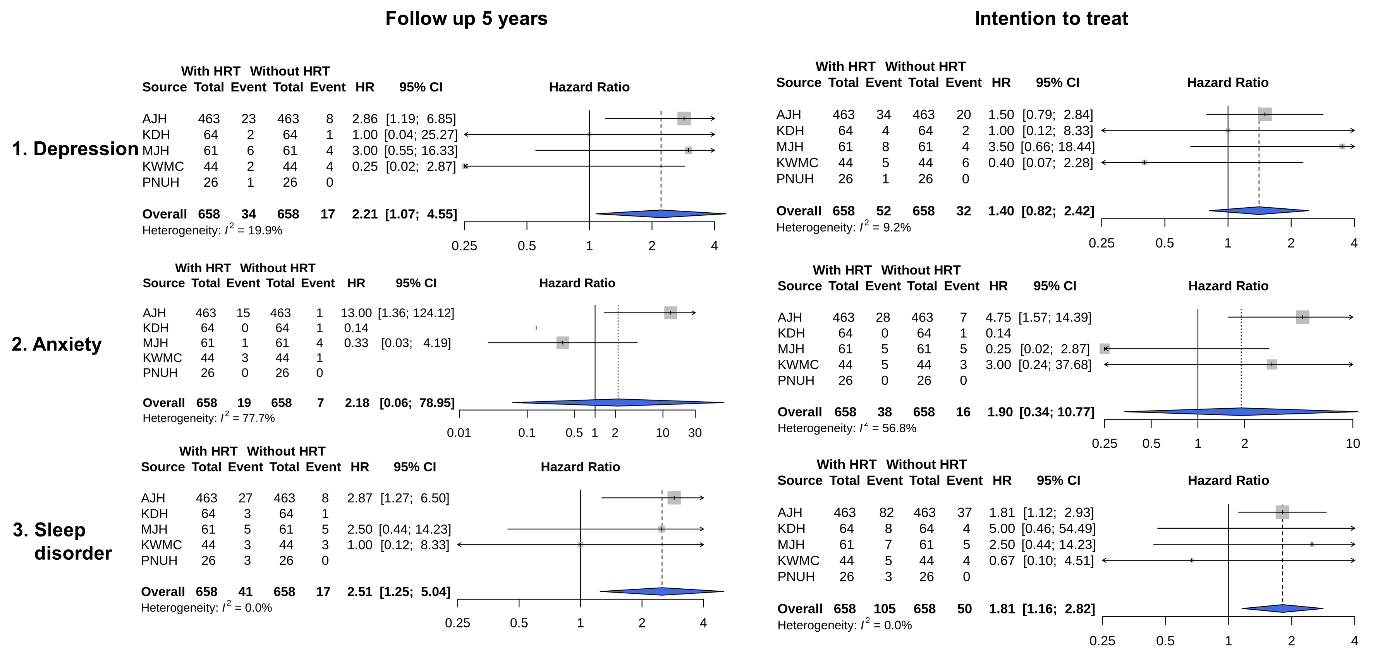

Supplement: Lee et al. supplementary material [file S0924933823024392sup001.docx]
